# Monocyte-derived transcriptomes explain the ineffectiveness of abatacept in rheumatoid arthritis

**DOI:** 10.1186/s13075-023-03236-y

**Published:** 2024-01-02

**Authors:** Takeshi Iwasaki, Ryu Watanabe, Hiromu Ito, Takayuki Fujii, Koichiro Ohmura, Hiroyuki Yoshitomi, Koichi Murata, Kosaku Murakami, Akira Onishi, Masao Tanaka, Shuichi Matsuda, Fumihiko Matsuda, Akio Morinobu, Motomu Hashimoto

**Affiliations:** 1 Department of Rheumatology and Clinical Immunology, Kyoto University Graduate School of Medicine, Kyoto, Japan; 2 Center for Genomic Medicine, Kyoto University Graduate School of Medicine, Kyoto, Japan; 3 Department of Clinical Immunology, Osaka Metropolitan University Graduate School of Medicine, Osaka, Japan; 4 Department of Orthopaedic Surgery, Kurashiki Central Hospital, Okayama, Japan; 5 Department of Advanced Medicine for Rheumatic Diseases, Kyoto University Graduate School of Medicine, Kyoto, Japan; 6 Department of Orthopaedic Surgery, Kyoto University Graduate School of Medicine, Kyoto, Japan; 7 Department of Rheumatology, Kobe City Medical Center General Hospital, Kobe, Japan; 8 Department of Immunology, Kyoto University Graduate School of Medicine, Kyoto, Japan; 9 Division of Clinical Immunology and Cancer Immunotherapy, Center for Cancer Immunotherapy and Immunobiology, Graduate School of Medicine, Kyoto University, Kyoto, Japan

**Keywords:** Rheumatoid arthritis, Abatacept, Multi-omics, Monocyte, TLR5, MYD88, IL17RA, OXPHOS

## Abstract

**Background:**

The biological mechanisms underlying the differential response to abatacept in patients with rheumatoid arthritis (RA) are unknown. Here, we aimed to identify cellular, transcriptomic, and proteomic features that predict resistance to abatacept in patients with RA.

**Methods:**

Blood samples were collected from 22 RA patients treated with abatacept at baseline and after 3 months of treatment. Response to treatment was defined by the European League Against Rheumatism (EULAR) response criteria at 3 months, and seven patients were classified as responders and the others as non-responders. We quantified gene expression levels by RNA sequencing, 67 plasma protein levels, and the expression of surface molecules (CD3, 19, and 56) by flow cytometry. In addition, three gene expression data sets, comprising a total of 27 responders and 50 non-responders, were used to replicate the results.

**Results:**

Among the clinical characteristics, the number of monocytes was significantly higher in the non-responders before treatment. Cell type enrichment analysis showed that differentially expressed genes (DEGs) between responders and non-responders were enriched in monocytes. Gene set enrichment analysis, together with single-cell analysis and deconvolution analysis, identified that Toll-like receptor 5 (TLR5) and interleukin-17 receptor A (IL17RA) pathway in monocytes was upregulated in non-responders. Hepatocyte growth factor (HGF) correlated with this signature showed higher concentrations in non-responders before treatment. The DEGs in the replication set were also enriched for the genes expressed in monocytes, not for the TLR5 and IL17RA pathway but for the oxidative phosphorylation (OXPHOS) pathway.

**Conclusions:**

Monocyte-derived transcriptomic features before treatment underlie the differences in abatacept efficacy in patients with RA. The pathway activated in monocytes was the TLR5 and IL17RA-HGF signature in the current study, while it was the OXPHOS pathway in the replication set. Elevated levels of HGF before treatment may serve as a potential biomarker for predicting poor responses to abatacept. These findings provide insights into the biological mechanisms of abatacept resistance, contributing valuable evidence for stratifying patients with RA.

**Supplementary Information:**

The online version contains supplementary material available at 10.1186/s13075-023-03236-y.

## Background

Rheumatoid arthritis (RA) is a chronic autoimmune and inflammatory disease that leads to joint destruction. Abatacept (CTLA4Ig) binds to CD80 and CD86 on antigen-presenting cells, thereby blocking the costimulatory interaction with CD28 on T cells and inhibiting its activation. This biological treatment is often considered when Phase I interventions, including the usage of methotrexate (MTX), do not sufficiently control disease activity of RA [1]. Most of the patients receive beneficial effects from abatacept; however, some exhibit poor responses to the therapy [2, 3], prompting extensive studies into factors influencing its outcome.

Numerous studies have investigated potential contributors to non-responsiveness. As the factors for non-responders, low anti-citrullinated peptide (ACPA) titer [4, 5], low rheumatoid factor (RF) titer [5, 6], lower number of CD19 positive cells [7], higher number of CD28 negative T cells [8], higher interferon signature [9], and shared-epitope (SE)-negative [10] have been reported. However, no biomarkers have been considered superior to ACPA/RF that are clinically useful and biologically interpretable. Consequently, there is an unmet need for further exploration concerning this critical issue.

Here, we aimed to identify cellular, transcriptomic, and proteomic features that predict responses or resistance to abatacept in a Japanese RA cohort as well as using public gene-expression data.

## Methods

### Study design

**Fig. 1:**
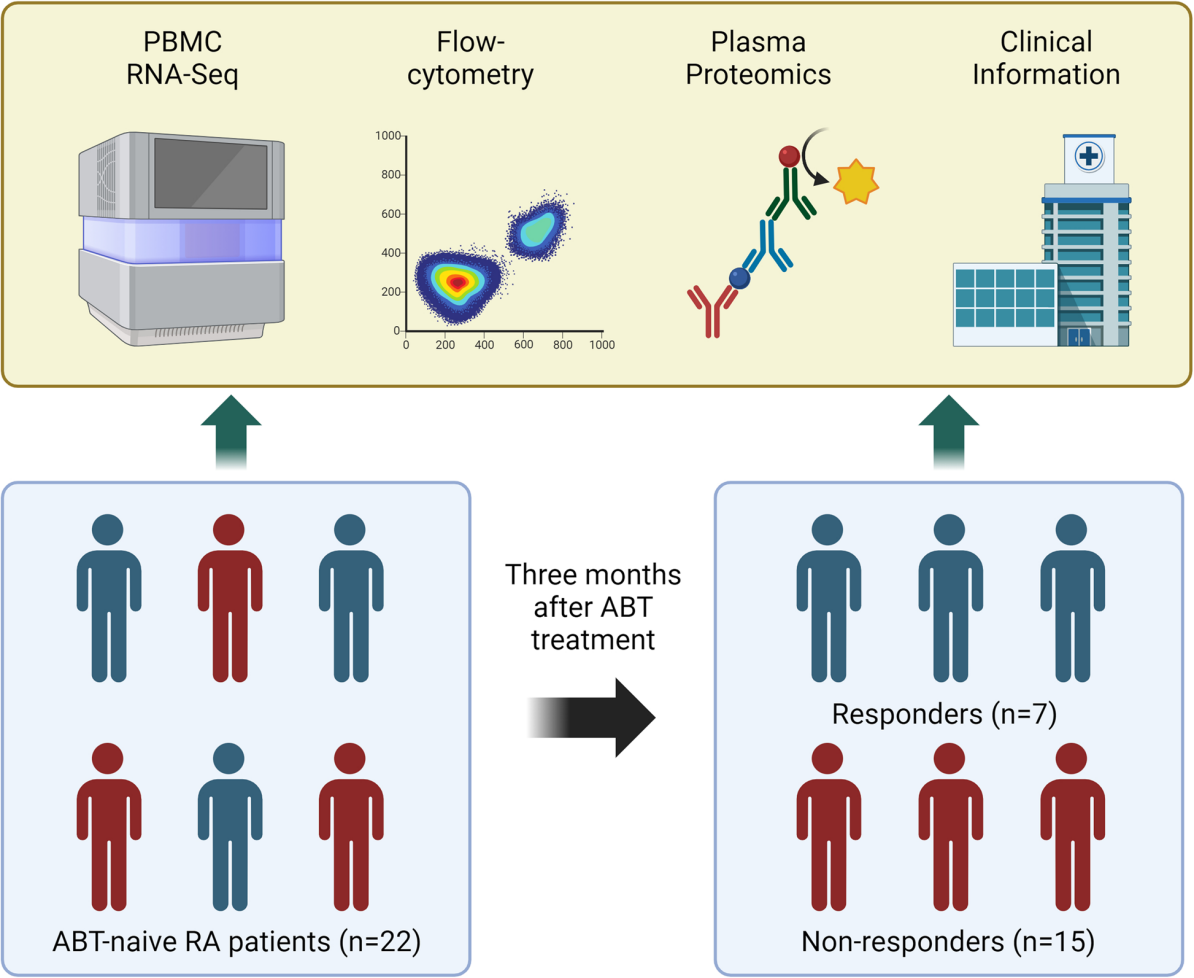
Overview of this study. PBMC, peripheral blood mononuclear cells; ABT, abatacept, RA. rheumatoid arthritis. Created with BioRender.com

We enrolled 22 RA patients who received abatacept therapy at the Center for Rheumatic Disease at Kyoto University Hospital. RA was diagnosed according to either the 1987 revised American College of Rheumatology classification criteria [11] or the 2010 America College of Rheumatology/European League Against Rheumatism (ACR/EULAR) criteria [12]. The overview of this study is shown in Fig. 1. Peripheral blood was collected from patients before and approximately 3 months after initiating abatacept treatment using heparin and ethylenediaminetetraacetic acid (EDTA) blood collection tubes.

### Clinical evaluation and response measure

Disease activity was evaluated using the Disease Activity Score 28-Erythrocyte sedimentation rate (DAS28-ESR) at every clinic visit. Clinical characteristics, including age, sex, body mass index (BMI), smoking history, duration of RA, the titers of RF, anti-cyclic citrullinated peptide (CCP) antibodies, anti-nuclear antibodies (ANA), ESR, C-reactive protein (CRP) and treatment profiles [the use of MTX and prednisolone (PSL)] before the initiation of treatment, and white blood cell count, blood differential count before and 3 months after the initiation of treatment, were obtained from medical records. The Steinblocker stage was assessed by rheumatologists. We classified patients using the European League Against Rheumatism (EULAR) response criteria [13] at 3 months after treatment initiation. Moderate and no responders were classified as non-responders, and the others as good responders.

### RNA sequencing, transcriptome analysis

RNA sequencing and quantification of gene expression were performed as described previously [14]. In short, RNA was extracted from peripheral blood mononuclear cells (PBMCs), and sequencing was conducted by HiSeq 2500 in the 150-bp paired-end mode. We trimmed reads by Cutadapt (ver1.1), aligned to the GRCh37 reference genome by STAR [15] (ver. 2.7.3a), counted gene expression by RSEM [16] (ver. 1.3.1), performed normalization using size factor implemented in DeSeq2 [17], and converted to count per million (CPM). The Wald test performed a differential gene expression analysis using DESeq2. Gene set enrichment analysis [18] was performed for 32,880 gene modules in MSigDB (ver 7.5) [19] with 10,000 permutations. We focused on the results with positive enrichment scores. As for the analysis of the replication data set, gene set enrichment analysis was performed by Metascape [20] with an input of 166 genes contributing to monocyte enrichment. Cell-type enrichment analysis was performed by WebCSEA [21] with the default setting. In both the current study and the replication dataset, the top 500 genes with the lowest *P* values were used as input since this platform accepts no more than 500 genes as input. This method outputs enrichment statistics for each cell type, along with a list of genes contributing to the enrichment of each cell type. The HLA-DRB1 allele was typed using HLA-HD (ver. 1.5.0) [22] and the shared epitope (SE) allele was classified as a previous report [23].

### Flow cytometry, and protein measurements

Flow cytometry and protein measurements were performed as described previously [14]. In short, we assessed the surface molecule expression by BD Canto™ II using the following antibodies obtained from BD Pharmingen: allophycocyanin-conjugated anti-CD56 (341025, NCAM16); antiCD3 (566683, OKT3), and V500-conjugated CD19 (561121, HIB19). We gated lymphocytes based on forward scatter (FSC) and side scatter (SSC) parameters and then calculated the percentage of each cell fraction in lymphocytes. The analysis was conducted using FlowJo software. The absolute number of each cell fraction in peripheral blood (× 10^6^/mL) was calculated using the percentage and an absolute number of lymphocytes measured by the hematology analyzer MEK-7300 (Nihon Kohden®). We measured 67 proteins in plasma using ProcartaPlex Human 15-plex, ProcartaPlex Human 49-plex, Human VCAM-1 Simplex, Human sICAM-1 Simplex, and Human sCGF Simplex with Bio-Plex 200 (BIO-RAD) according to the manufacturer’s instructions. These assays were selected during the study's design phase to maximize the number of measurable proteins through a multiplex system.

### Replication data set

We searched the gene expression data concerning abatacept response in gene expression omnibus (GEO) as much as possible in January 2023. We utilized three datasets; the breakdown is shown in Table S1. In each case, responders and non-responders were defined by the available information. As for GSE78068 [24], responders were defined by remission according to the Clinical Disease Activity Index (CDAI) at six months of therapy. As for GSE68215 [25], non-responders were defined as moderate / no response according to EULAR criteria at six months of therapy. As for GSE172188 [26], non-responders were defined as moderate / no response according to EULAR criteria at 16 weeks of therapy. We performed an association test using limma (ver. 3.46.0) [27] in each cohort and meta-analyzed the results by MetaVolcanoR (ver. 1.14). The scripts to analyze each data set and meta-analysis are provided in the GitHub repository: https://github.com/takeshiiwasaki/abatacept. We defined the oxidative phosphorylation (OXPHOS) score as the mean expression of 200 genes included in “HALLMARK_OXIDATIVE_PHOSPHORYLATION” in MSigDB (ver. 7.5) [19].

### Single-cell analysis and deconvolution analysis

To elucidate the cellular origin of the detected module, we used the single-cell data of PBMCs [28] and synovium [29] from RA patients. The process of annotating cell types was documented in the provided GitHub repository: https://github.com/takeshiiwasaki/abatacept. We performed a deconvolution analysis using CIBERSORTx [30] to estimate the expression of genes in monocytes. As a reference, we used the single-cell RNA expression data of PBMCs from RA patients [28].

### Ethics approval and consent to participate

The present study was performed following the Helsinki Declaration and was approved by the Kyoto University Graduate School and Faculty of Medicine Ethics Committee (approval number: G0511). Written informed consent to participate in the present study and publish the results obtained was provided by all enrolled patients.

## Results

### Comparison of clinical characteristics of responders and non-responders

Twenty-two RA patients were enrolled in this study (Fig. 1). Seven (31.8%) patients had a good response to abatacept, while 15 (68.2%) did not. The clinical characteristics of patients at the treatment initiation are summarized in Table 1. There was no difference in baseline clinical characteristics between responders and non-responders (*P* > 0.05). However, when we investigated cellular phenotype, we found a higher number of monocytes (*P* = 0.02) and a lower number of CD3^+^ cells with suggestive significance (*P* = 0.09) in non-responders before treatment. After 3 months of treatment, there remains a tendency of a higher number of monocytes and a lower number of CD3^+^ cells in non-responders (Table S2) (*P* = 0.09, 0.03, respectively).

**Table 1:** Clinical and cellular characteristics of responders and non-responders

	**Responders**	**Non-responders**	**OR (95% CI)**	***P***^***a***^
Total	7	15		
Age	68.0 (60.6–73.5)	66.0 (64.5–70.5)	ND	0.97
Female (%)	7 (100)	12 (80)	0.00 (0.00–5.3)	0.52
BMI	19.5 (18.9–23.0)	24.3 (22.2–26.0)	ND	0.09
Smoking history (%)	0 (0)	3 (23.4)	Inf (0.15–Inf)	0.52
Duration of RA (years)	11.1 (7.1–22.7)	13.0 (5.6–19.7)	ND	0.78
CRP (mg/dL)	0.6 (0.4–1.3)	0.7 (0.3–1.8)	ND	0.67
ESR (mm/h)	30.0 (24.0–39.5)	35.0 (18.0–49.0)	ND	0.80
RF positivity (%)	6 (85.7)	13 (86.7)	1.08 (0.02–24.8)	1.00
Anti-CCP positivity (%)	7 (100)	11 (73.3)	0 (0–3.2)	0.26
MTX usage (%)	3 (42.9)	9 (60.0)	1.94 (0.23–18.5)	0.65
MTX dosage^†^ (mg/week)	0.0 (0.0–6.0)	6.0 (0.0–8.0)	ND	0.46
PSL usage (%)	2 (28.6)	8 (53.3)	2.7 (0.3–37.4)	0.38
PSL dosage^†^ (mg/day)	0.0 (0.0–2.5)	6.0 (0.0–8.0)	ND	0.46
Previous b/tsDMARDs usage (%)	3 (42.9)	6 (40.0)	0.89 (0.10–8.43)	1.00
cDMARDs usage (%)	4 (57.1)	11 (73.3)	1.99 (0.20–19.2)	0.63
SE (%)	11 (73.3)	5 (71.4)	1.1 (0.1–11.2)	1.00
DAS28-ESR (0 months)	4.4 (4.0–4.6)	5.3 (3.5–5.6)	ND	0.36
DAS28-ESR (3 months)	2.8 (2.3–3.0)	3.4 (2.9–4.2)	ND	0.07
Cellular phenotype (× 10^3^/μL)				
White blood cell	5.43 (5.19–7.24)	7.18 (5.15–10.6)	ND	0.62
Neutrophil	3.75 (3.12–5.12)	4.92 (3.29–8.86)	ND	0.53
Lymphocyte	1.40 (1.10–1.70)	1.20 (1.00–1.30)	ND	0.32
CD3 + cell	1.02 (0.77–1.16)	0.72 (0.49–0.88)	ND	0.09
CD19 + cell	0.19 (0.10–0.20)	0.11 (0.07–0.24)	ND	0.78
CD56 + cell	0.23 (0.19–0.28)	0.22 (0.16–0.32)	ND	0.83
Monocyte	0.28 (0.23–0.30)	0.43 (0.31–0.55)	ND	0.02
Eosinophil	0.13 (0.04–0.21)	0.10 (0.05–0.18)	ND	0.86
Basophil	0.03 (0.02–0.06)	0.07 (0.03–0.08)	ND	0.62

Data were described as medians (interquartile range (IQR)) for continuous variables and numbers (percentages) for categorical variables

*OR* Odds ratio, *ND* No data, *Inf* Infinity, *BMI* Body mass index, *CRP* C-reactive protein, *ESR* Erythrocyte sedimentation rate, *RF* Rheumatoid factor, *CCP* Cyclic citrullinated peptide, *PSL* Prednisolone, *MTX* Methotrexate, *b/tsDMARDs* biologic/targeted synthetic disease-modifying antirheumatic drugs, including etanercept, tocilizumab, infliximab, adalimumab, golimumab, *cDMARDs* conventional DMARDs, including methotrexate, bucillamine, salazosulfapyridine, tacrolimus, cyclosporine, mizoribine, leflunomide, and iguratimod

^a^The Mann–Whitney *U* test was used for continuous variables and Fisher’s exact test for categorical variables, †Calculated from all samples regardless of the corresponding drug usage

### Relationship between gene expression and response

**Fig. 2:**
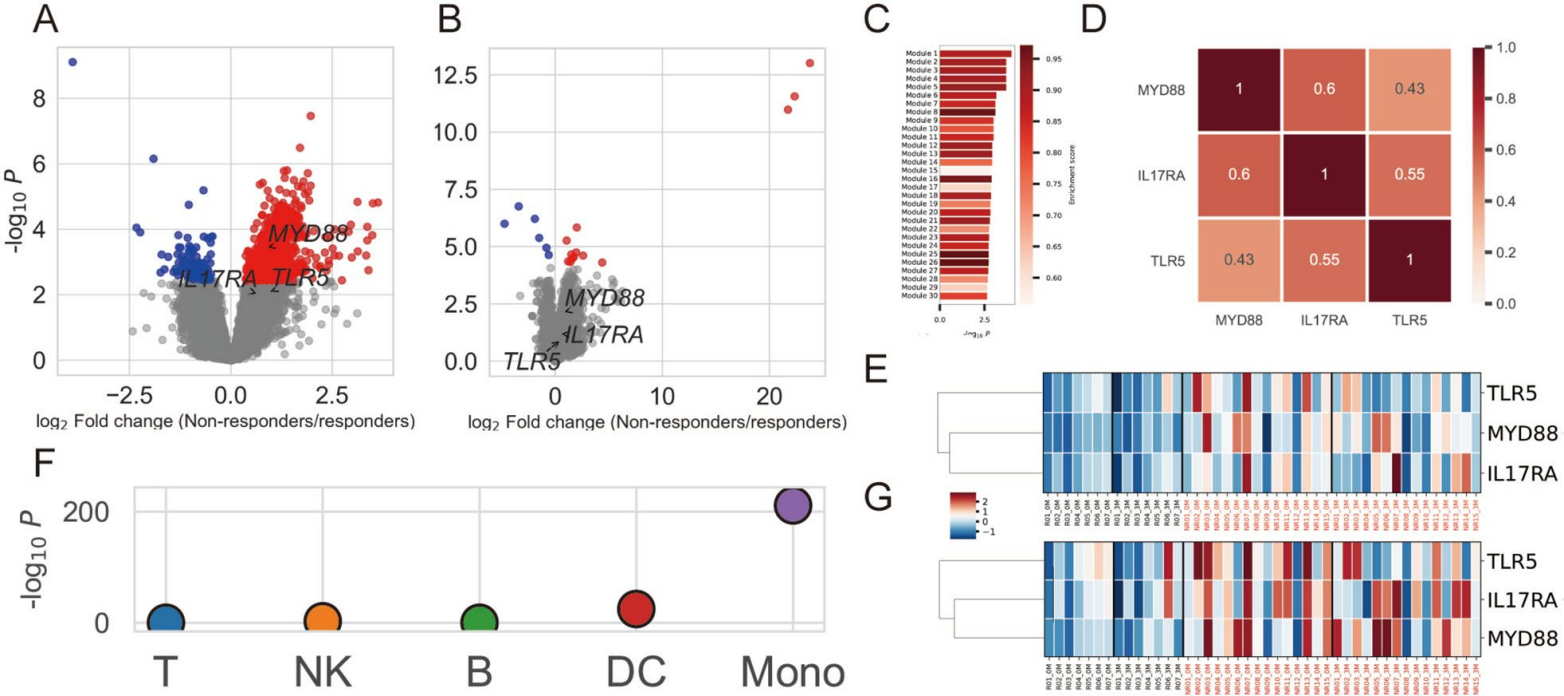
Transcriptomic analysis of the current study. **A**, **B** Volcano plots showing differentially expressed genes between responders and non-responders before treatment (**A**) and three months after treatment (**B**). **C** Results of gene set enrichment analysis before treatment. **D** Correlation between each gene’s expressions. The number in each box represents the Pearson's correlation coefficient. **E** Expression was standardized across all samples, and Z scores were shown. Sample names beginning with “R” indicate responders, “NR” indicates non-responders; sample names ending with “0 M” indicate pre-treatment, and “_3M” indicates 3 months post-treatment. **F** Results of cell type enrichment analysis before treatment. T, T cell; NK, NK cell; B. B cell; DC. dendritic cell; Mono, monocyte. **G** Estimated gene expression in monocytes. As in **E**, *Z* scores are shown

We performed a differential gene expression analysis between responders and non-responders before treatment to identify transcriptomic features that predict response to abatacept. We identified 952 differentially expressed genes (DEGs) associated with treatment responses (false discovery rate; FDR < 0.05) (Fig. 2A, Table S3). After 3 months, the number of DEGs decreased to 19 (Fig. 2B, Table S4). We performed a gene-set enrichment analysis [16] using the transcriptomic data before treatment to characterize transcriptomic differences. As a result, we found the strongest enrichment in “module 1” (*P* = 1.0 × 10^−4^, Enrichment score = 0.89) (Fig. 2C, Table S5). We interpreted this enrichment based on the current transcriptomic data and existing literature (Supplementary Data S1) and determined it was attributed to the upregulation of Toll-like receptor 5 (TLR5), myeloid differentiation primary response protein 88 (MYD88), and interleukin-17 receptor A (IL17RA) in non-responders. These three genes are significantly correlated with each other in 44 specimens (22 samples with two-time points) (Fig. 2D, Table S6) and exhibited higher expression levels in non-responders both before treatment as well as 3 months after treatment (Fig. 2E).

Next, to detect the cellular origin of the upregulated transcriptomic feature in non-responders, we performed a cell-type enrichment analysis. The results showed the strongest enrichment on monocytes among all the major immune cell types in PBMCs (*P* = 8.7 × 10^−212^, Fig. 2F). When we estimated the expression of TLR5, MYD88, and IL17RA in monocytes by deconvolution analysis, the non-responders had higher expression before treatment initiation as well as 3 months after treatment (Fig. 2G), MYD88 showing significant upregulation in non-responders before treatment as well as 3 months after treatment (Table S7).

### Cellular origin of TLR5, MYD88, and IL17RA

**Fig. 3:**
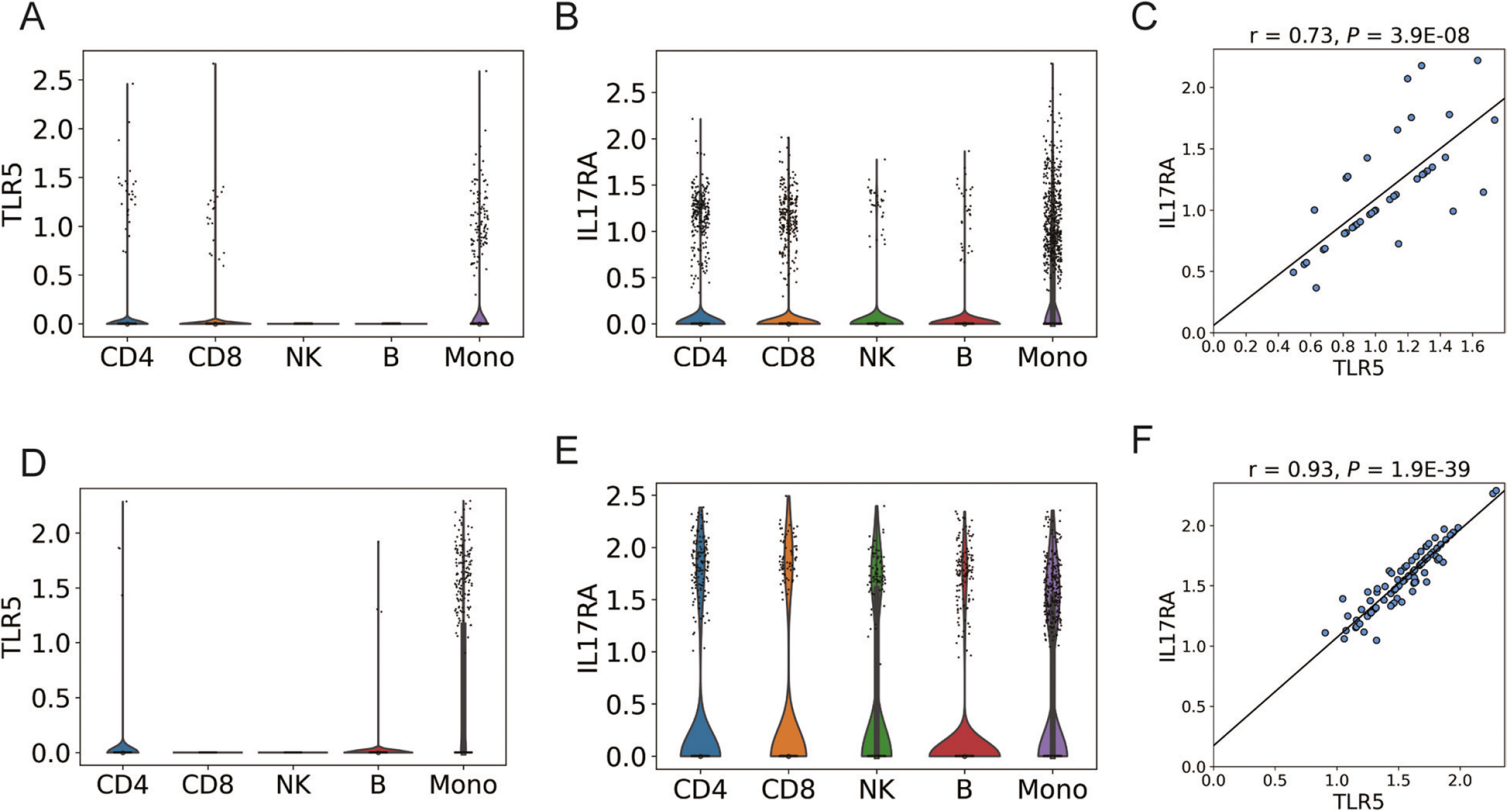
Single-cell analysis of specimens derived from patients with rheumatoid arthritis. Violin plots comparing the expression of TLR5 (**A**) and IL17RA (**B**) in peripheral blood from RA patients. **C** A scatter plot of IL17RA and TLR5 expression in TLR5 + IL17RA + monocytes in peripheral blood in RA patients. The Pearson’s correlation coefficient (*r*), and the *P*-value calculated from linear regression are shown. **D**, **E** Violin plots comparing the expression of TLR5 (**D**) and IL17RA (**E**) in synovium from RA patients. **F** A scatter plot of IL17RA and TLR5 expression in TLR5^+^IL17RA^+^ monocytes in synovium cells in RA patients. Pearson’s correlation coefficient (*r*), and the *P*-value calculated from linear regression are shown. CD4, CD4 + T cell; CD8, CD8 + T cell; NK, NK cell; B. B cell; Mono. monocyte

To further investigate the cellular origin of the TLR5, MYD88, and IL17RA, we analyzed single-cell data from PBMCs of RA patients. First, we found that TLR5, as a representative gene of TLR5 and MYD88, and IL17RA are mainly expressed by monocytes (Fig. 3A, B). Furthermore, we identified a subset of monocytes that co-express TLR5 and IL17RA (42 out of 1298 monocytes; 3.2%), with their expression showing a correlation (Fig. 3C). Hereafter, we will refer to these monocytes as “TLR5^+^IL17RA^+^ monocytes.”

We also validated the cellular origin of TLR5, MYD88, and IL17RA in RA synovium using single-cell data. Consistent with the findings in PBMCs, monocytes were the immune cell type with the highest expression of TLR5 and IL17RA (Fig. 3D, E). We also found TLR5^+^IL17RA^+^ monocytes, and their expression exhibited a strong correlation (Fig. 3F). The proportion of TLR5^+^IL17RA^+^ monocytes among monocytes was 14.5% (87 out of 602 cells), which was 5.1-fold higher than that of PBMCs (*P* = 8.2 × 10^−18^, Fisher’s exact test).

### Identifying proteins associated with the detected signature and treatment response

**Fig. 4:**
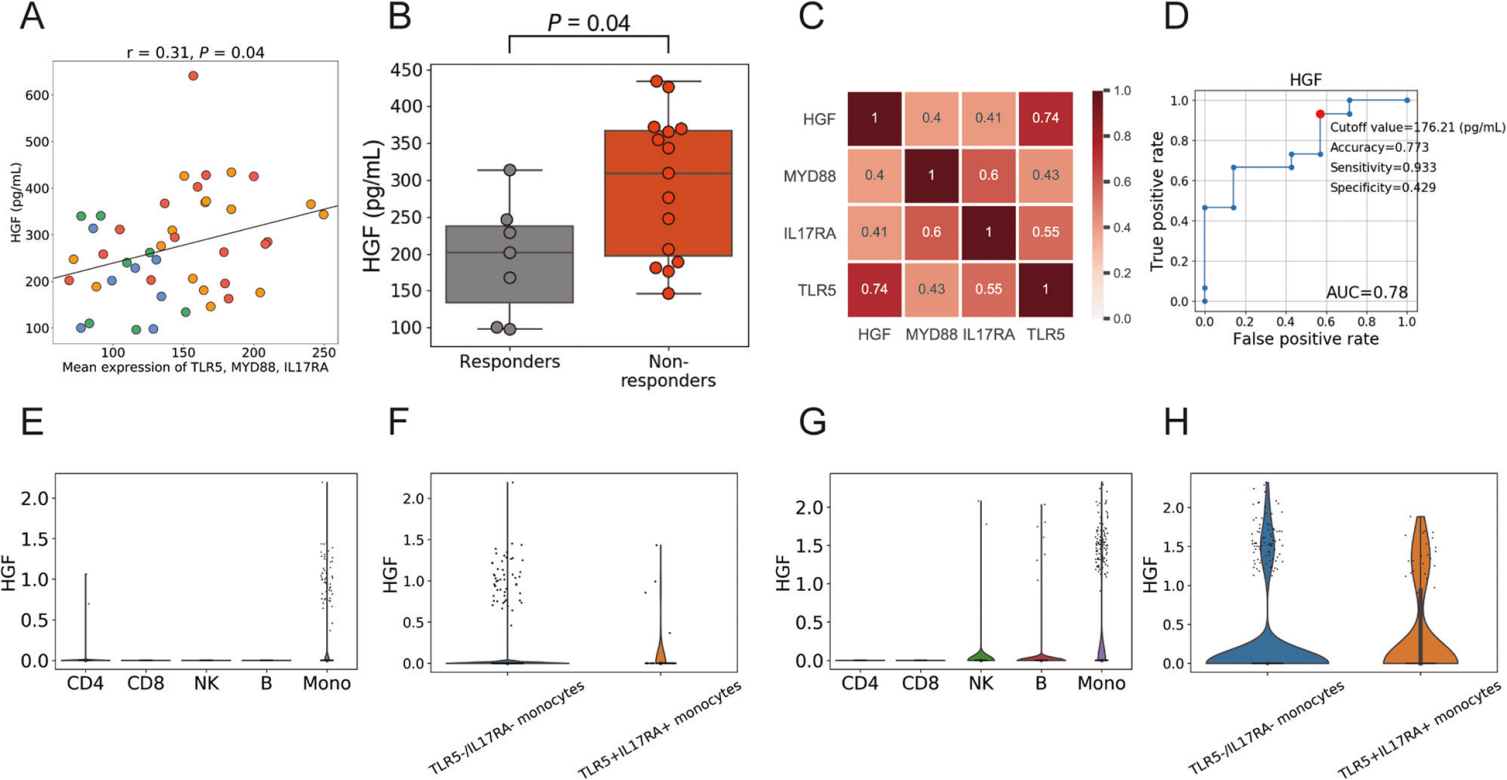
Identification of HGF as an alternative biomarker of TLR5-MYD88 and IL17RA pathway. **A** Before treatment of responders (blue) and non-responders (orange), three months after treatment of responders (green) and non-responders (red). The Pearson's correlation coefficient (*r*), and the *P*-value calculated from linear regression are shown. **B** Comparison of HGF levels before treatment data. The *P*-value calculated by the Mann–Whitney U test is shown. **C** Correlation between each gene’s expression. The number in each box represents Pearson’s correlation coefficient. **D** ROC curves for no response to abatacept. **E**–**H** Expression of HGF in immune cells using single-cell data from peripheral blood (**E**, **F**) and synovium (**G**, **H**). CD4, CD4 + T cell; CD8. CD8 + T cell; NK. NK cell; B. B cell; Mono, monocyte

To identify the protein associated with this transcriptomic signature and treatment response, we calculated the associations between the mean expression of the three genes (TLR5, MYD88, and IL17RA) and the 67 proteins measured by multiplex immunoassay (see Methods) using 44 specimens (22 individuals × 2 time points). Among the 67 protein levels, C-X-C Motif Chemokine Ligand 10 (CXCL10), IL-8, hepatocyte growth factor (HGF), and IL-20 showed significant association with the expression of this module (Fig. 4A, Table S8). Among the four proteins, we found HGF was significantly higher in non-responders before treatment (*P* = 0.04, Fig. 4B, Table S9). We confirmed that the expression of the HGF was significantly correlated with those of TLR5, MYD88, and IL17RA using transcriptomic data derived from 44 specimens (Fig. 4C, Table S6). To confirm the potential to predict the response to abatacept by measuring the levels of HGF before treatment initiation, we performed a receiver-operating-characteristic (ROC) analysis. The area under the ROC curve (AUC) for HGF was 0.78, and the most accurate cut-off level was 176.21 pg/mL (Fig. 4D). These results suggest that the level of HGF protein is associated with the detected transcriptomic feature as well as a good predictor of response.

We then investigated the cellular origin of HGF using the same single-cell data. We found that monocytes primarily express HGF (Fig. 4E). We further compared the expression of HGF between TLR5^−^/IL17RA^−^ monocytes and TLR5^+^IL17RA^+^ monocytes (Fig. 4F). As a result, we observed an enrichment of HGF expression in TLR5^+^IL17RA^+^ monocytes (4 out of 42 cells vs. 52 out of 9469 cells; 17.4-fold enrichment, *P* = 1.4 × 10^−4^, Fisher’s exact test). We did the same analysis using the synovium single-cell data. Consistent with the result in PBMCs, HGF was expressed by monocytes (Fig. 4G) and was enriched in TLR5^+^IL17RA^+^ monocytes compared to TLR5^−^/IL17RA^−^ monocytes (Fig. 4H) (23 out of 87 cells vs. 87 out of 515 cells; 2.4-fold enrichment, *P* = 1.1 × 10^−4^).

When we calculated the levels of HGF and the number of monocytes in the current data (22 individuals × 2 time points), we observed a positive correlation (Fig. S1), suggesting the number of monocytes in peripheral blood could be an alternative biomarker for HGF. Furthermore, based on several previous literatures indicating the association between HGF and bone damage [31, 32], we analyzed the relationship between the Steinblocker stage and the HGF levels before treatment, but found no significant association (Fig. S2).

### Replication of the current results

**Fig. 5:**
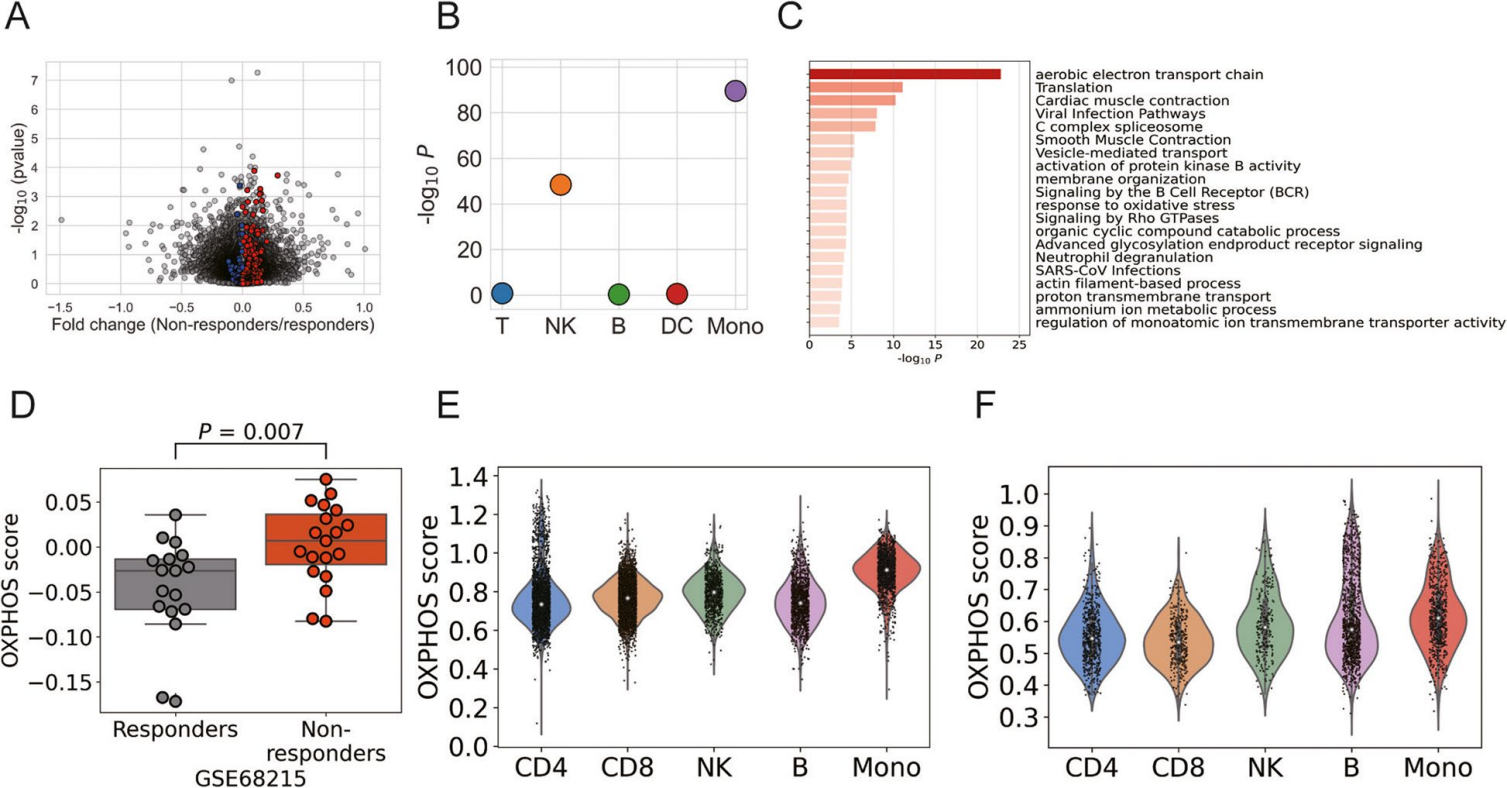
Transcriptomic analysis of the replication set. **A** Transcriptomic association study between responders vs. non-responders (meta-analysis of three datasets). Genes related to the OXPHOS pathway are color-coded(red: upregulated in non-responders, blue: downregulated in non-responders). **B** Results of cell type enrichment analysis. **C** Enrichment analysis of genes contributing to monocyte enrichment. **D** The OXPHOS scores in the GSE68215 data set. The *P*-value calculated by the Mann–Whitney U test is shown. **E**, **F** The OXPHOS scores in immune cells in peripheral blood (**E**) and in synovium tissue (**F**) in patients with RA. CD4, CD4 + T cell; CD8, CD8 + T cell; NK, NK cell; B, B cell; Mono, monocyte

We assessed the obtained results across various datasets, compiling a gene expression dataset related to abatacept efficacy to maximize our sample size. Through meta-analysis of the three data sets (Table S1), we identified 839 genes exhibiting nominal significance (*P* < 0.05, Fig. 5A, Table S10). As for TLR5, MYD88, and IL17RA, they did not show any significant differences between responders and non-responders (*P* > 0.05). However, differentially expressed genes were notably enriched for those expressed in monocytes (*P* = 2.9 × 10^−90^) (Fig. 5B). The set of 166 genes that contribute to monocyte enrichment (Table S11) demonstrated enrichment for genes involved in the aerobic respiration / OXPHOS pathway (Fig. 5C, Table S12). To quantify this difference, we calculated the mean expression of the OXPHOS pathway (designated as the “OXPHOS score”) and compared it between responders and non-responders in each study. A significant increase in OXPHOS score in non-responder was observed in the GSE68215 dataset (Fig. 5D), while there was no statistical significance in all the other datasets (Fig. S3). Finally, using single-cell datasets, we investigated the cellular source of the OXPHOS signature within immune cells. We found that the highest expression of the OXPHOS signature was observed in monocytes, both within PBMCs (Fig. 5E) and within the synovium (Fig. 5F).

## Discussion

We have identified that HGF-producing TLR5^+^IL17RA^+^ monocytes play critical roles in the ineffectiveness of abatacept in our study. Although this finding was not replicated in the replication set, we have demonstrated that distinct monocyte-derived transcriptome features before treatment underlie differences in the efficacy of abatacept. Given that abatacept's mechanism of action involves inhibiting T-cell activation, the finding that the presence of activated monocytes determines the efficacy of abatacept is understandable.

The pathway activated in monocytes was the TLR5 and IL17RA to HGF pathway in the current study. According to the single-cell data derived from PBMCs and synovium from RA patients, there were TLR5^+^ IL17RA^+^ monocytes, and HGF expression was enriched in that cell type. IL17A is known to modulate both expression of TLR5 and IL17RA [33, 34]. Expression of IL17A did not show a significance difference between responders and non-responders (Tables S3, S4). This lack of significance may be attributed to the limited statistical power arising from the low expression of IL17A (Tables S3, S4). Therefore, increasing RNA sequencing coverage could potentially capture the variations in IL17A expression and unravel the intricate relationship between its expression, IL17RA, and TLR5. Furthermore, we noted an association of HGF with TLR5, MYD88, and IL17RA signatures. HGF is associated with osteoclastogenesis in Collagen-induced arthritis (CIA) mice [31] and radiographic damage in RA patients [32]. Consequently, our findings gain significance by connecting abatacept treatment resistance to HGF's acknowledged pathological role in RA.

The OXPHOS is a metabolic pathway to produce adenosine triphosphate (ATP) in mitochondria and is involved in many immune cell functions [35] and dysregulation of this pathway is related to multiple autoimmune diseases, including RA [36]. It is reported that increased levels of this signature in monocytes in RA [37]. Our study introduces the intriguing possibility of utilizing this OXPHOS signature within monocytes as a tool to stratify RA patients.

This study has the limitation of a relatively small sample size in the current data. Especially, the re-evaluation of the cut-off level for HGF (Fig. 4D) is warranted in an independent and larger cohort. Nevertheless, our study yields valuable insights with direct clinical implications. Firstly, it is imperative to exercise caution when dealing with RA patients exhibiting elevated HGF levels, as this has been linked to poor responses not only to abatacept but also to TNF inhibitors [14] and the advancement of radiographic damage [34]. Although we couldn’t identify a significant association between HGF and bone damage in the current dataset (Fig. S2), further analysis is warranted to explore the potential effect of HGF on long-term bone damage. Secondly, our findings suggest an approach for patients whose monocytes exhibit activation of the TLR5 and IL17RA to the HGF pathway or the OXPHOS pathway. In such cases, exploring treatment options targeting innate immune cells’ modulation could offer a promising alternative strategy. Interventions such as anti-IL-6 or anti-GM-CSF or JAK inhibitor [38, 39] may be particularly effective in these scenarios, potentially offering enhanced clinical outcomes.

## Conclusions

We have demonstrated that monocyte-derived transcriptomic features before treatment underlie the differences in abatacept efficacy. In the current study, the pathway activated in monocytes was the TLR5 and IL17RA to HGF pathway, while in the replication set, it was the OXPHOS pathway. The levels of HGF before treatment initiation may serve as a potential biomarker for predicting poor responses to abatacept.

### Supplementary Information

**Additional file 1:** **Supplementary Data S1.** Interpretation of module 1 enrichment

**Additional file 2: Fig. S1.** Correlation between the number of monocytes and the level of HGF calculated in 44 specimens (22 individuals × 2 time points). The vertical line represents the number of monocytes in the peripheral blood (×10^3^/μL). The dots indicate the specimens before treatment of responders (blue) and non-responders (orange), 3 months after treatment of responders (green) and non-responders (red). The Pearson's correlation coefficient (r), and the *P*-value calculated from linear regression are shown.

**Additional file 3: Fig. S2.** Correlation between the Steinblocker stage and the levels of HGF before treatment calculated in 22 individuals. The Pearson's correlation coefficient (r), and the *P*-value calculated from linear regression are shown.

**Additional file 4: Fig. S3.** Box plots comparing the OXPHOS scores between responders and non-responders in GSE78068 (A), GSE172188 (B), and the current study (C). The *P*-values calculated by the Mann-Whitney U test are shown.

**Additional file 5: Table S1.** Breakdown of replication sets. **Table S2.** Cellular characteristics of responders & non-responders after three months of treatment. **Table S3.** Results of an association analysis of gene expression between non-responders and responders (before treatment). **Table S4.** Results of an association analysis of gene expression between non-responders and responders (three months after treatment). **Table S5.** Results of gene set enrichment analysis between non-responders and responders. **Table S6.** Correlation between each gene's expression calculated from 44 specimens. **Table S7.** Association test of estimated expression in monocytes between responders and non-responders. **Table S8.** Results of an association analysis between mean expression of TLR5,MYD88 and IL17RA and 67 proteins. **Table S9.** Results of an association analysis of protein levels between responders and non-responders before treatment. **Table S10. **Results of meta-analysis of replication sets. **Table S11. **List of 166 genes that shows significant difference between responders and non-responders in the replication set as well as contribute to monocyte enrichment. **Table S12.** Results of gene set enrichment analysis of the 166 genes, shown in Supplementary Table S11.

## Data Availability

The gene expression dataset supporting the conclusions of this article is available in the Zenodo repository, 10.5281/zenodo.8250013. These data can be downloaded without restriction. Codes about critical points were deposited in https://github.com/takeshiiwasaki/abatacept. Other data and codes are available from the corresponding authors upon reasonable request.

## Abbreviations

RA: Rheumatoid arthritis
EULAR: European League Against Rheumatism
DEGs: Differentially expressed genes
TLR5: Toll-like receptor 5
MYD88: Myeloid differentiation primary response 88
IL17RA: Interleukin-17 receptor A
HGF: Hepatocyte growth factor
OXPHOS: Oxidative phosphorylation
AUC: Area under the curve
TNF: Tumor necrosis factor

## References

[1] Smolen JS, Landewé RBM, Bergstra SA, Kerschbaumer A, Sepriano A, Aletaha D (2023). EULAR recommendations for the management of rheumatoid arthritis with synthetic and biological disease-modifying antirheumatic drugs: 2022 update. Ann Rheum Dis.

[2] Rubbert-Roth A, Enejosa J, Pangan AL, Haraoui B, Rischmueller M, Khan N (2020). Trial of upadacitinib or abatacept in rheumatoid arthritis. N Engl J Med.

[3] Genovese MC, Schiff M, Luggen M, Becker JC, Aranda R, Teng J (2008). Efficacy and safety of the selective co-stimulation modulator abatacept following 2 years of treatment in patients with rheumatoid arthritis and an inadequate response to anti-tumour necrosis factor therapy. Ann Rheum Dis.

[4] Sokolove J, Schiff M, Fleischmann R, Weinblatt ME, Connolly SE, Johnsen A (2016). Impact of baseline anti-cyclic citrullinated peptide-2 antibody concentration on efficacy outcomes following treatment with subcutaneous abatacept or adalimumab: 2-year results from the AMPLE trial. Ann Rheum Dis.

[5] Gottenberg JE, Courvoisier DS, Hernandez MV, Iannone F, Lie E, Canhão H (2016). Brief report: association of rheumatoid factor and anti-citrullinated protein antibody positivity with better effectiveness of abatacept: results from the Pan-European registry analysis. Arthritis Rheumatol.

[6] Okazaki S, Watanabe R, Harigae H, Fujii H (2020). Better retention of abatacept is associated with high rheumatoid factor: a five-year follow-up study of patients with rheumatoid arthritis. Tohoku J Exp Med.

[7] Gazeau P, Devauchelle-Pensec V, Pochard P, Pers JO, Saraux A, Renaudineau Y (2016). Abatacept efficacy in rheumatoid arthritis is dependent upon baseline blood B-cell levels. Rheumatology (Oxford).

[8] Scarsi M, Ziglioli T, Airo P (2011). Baseline numbers of circulating CD28-negative T cells may predict clinical response to abatacept in patients with rheumatoid arthritis. J Rheumatol.

[9] Yokoyama-Kokuryo W, Yamazaki H, Takeuchi T, Amano K, Kikuchi J, Kondo T (2020). Identification of molecules associated with response to abatacept in patients with rheumatoid arthritis. Arthritis Res Ther.

[10] Oryoji K, Yoshida K, Kashiwado Y, Tanaka K, Mizuki SI, Tsukamoto H (2018). Shared epitope positivity is related to efficacy of abatacept in rheumatoid arthritis. Ann Rheum Dis.

[11] Frank A, Steven E, Daniel B, Dennis M, James F, Norman C (1988). The American Rheumatism Association 1987 revised criteria for the classification of rheumatoid arthritis. Arthritis Rheum.

[12] Aletaha D, Neogi T, Silman AJ, Funovits J, Felson DT, Bingham CO (2010). 2010 Rheumatoid arthritis classification criteria: An American College of Rheumatology/European League Against Rheumatism collaborative initiative. Ann Rheum Dis.

[13] Van Gestel AM, Prevoo MLL, Van ’T Hof MA, Van Rijswijk MH, Van De Putte LBA, Van Riel PLCM. Development and validation of the European League Against Rheumatism response criteria for rheumatoid arthritis. Comparison with the preliminary American College of Rheumatology and the World Health Organization/International League Against Rheumatism Criteria. Arthritis Rheum 1996;39:34–40. 10.1002/art.1780390105 8546736

[14] Iwasaki T, Watanabe R, Ito H, Fujii T, Okuma K, Oku T (2022). Dynamics of Type I and Type II Interferon Signature Determines Responsiveness to Anti-TNF Therapy in Rheumatoid Arthritis. Front Immunol.

[15] Dobin A, Davis CA, Schlesinger F, Drenkow J, Zaleski C, Jha S (2013). STAR: Ultrafast universal RNA-seq aligner. Bioinformatics.

[16] Li B, Dewey CN (2011). RSEM: Accurate transcript quantification from RNA-Seq data with or without a reference genome. BMC Bioinformatics.

[17] Love MI, Huber W, Anders S (2014). Moderated estimation of fold change and dispersion for RNA-seq data with DESeq2. Genome Biol.

[18] Subramanian A, Tamayo P, Mootha VK, Mukherjee S, Ebert BL, Gillette MA (2005). Gene set enrichment analysis: a knowledge-based approach for interpreting genome-wide expression profiles. Proc Natl Acad Sci U S A.

[19] Liberzon A, Subramanian A, Pinchback R, Thorvaldsdóttir H, Tamayo P, Mesirov JP (2011). Molecular signatures database (MSigDB) 3.0. Bioinformatics.

[20] Zhou Y, Zhou B, Pache L, Chang M, Khodabakhshi AH, Tanaseichuk O (2019). Metascape provides a biologist-oriented resource for the analysis of systems-level datasets. Nat Commun.

[21] Dai Y, Hu R, Liu A, Cho KS, Manuel AM, Li X (2022). WebCSEA: web-based cell-type-specific enrichment analysis of genes. Nucleic Acids Res.

[22] Kawaguchi S, Higasa K, Shimizu M, Yamada R, Matsuda F (2017). HLA-HD: An accurate HLA typing algorithm for next-generation sequencing data. Hum Mutat.

[23] Terao C, Ohmura K, Kochi Y, Ikari K, Maruya E, Katayama M (2011). A large-scale association study identified multiple HLA-DRB1 alleles associated with ACPA-negative rheumatoid arthritis in Japanese subjects. Ann Rheum Dis.

[24] Nakamura S, Suzuki K, Iijima H, Hata Y, Lim CR, Ishizawa Y (2016). Identification of baseline gene expression signatures predicting therapeutic responses to three biologic agents in rheumatoid arthritis: a retrospective observational study. Arthritis Res Ther.

[25] Derambure C, Dzangue-Tchoupou G, Berard C, Vergne N, Hiron M, D’Agostino MA (2017). Pre-silencing of genes involved in the electron transport chain (ETC) pathway is associated with responsiveness to abatacept in rheumatoid arthritis. Arthritis Res Ther.

[26] Triaille C, Durez P, Sokolova T, Tilman G, de MéricBellefon L, Galant C (2021). Common transcriptomic effects of abatacept and other DMARDs on rheumatoid arthritis synovial tissue. Front Immunol.

[27] Ritchie ME, Phipson B, Wu D, Hu Y, Law CW, Shi W (2015). limma powers differential expression analyses for RNA-sequencing and microarray studies. Nucleic Acids Res.

[28] Zhang B, Zhang Y, Xiong L, Li Y, Zhang Y, Zhao J (2022). CD127 imprints functional heterogeneity to diversify monocyte responses in inflammatory diseases. J Exp Med.

[29] Zhang F, Wei K, Slowikowski K, Fonseka CY, Rao DA, Kelly S (2019). Defining inflammatory cell states in rheumatoid arthritis joint synovial tissues by integrating single-cell transcriptomics and mass cytometry. Nat Immunol.

[30] Newman AM, Steen CB, Liu CL, Gentles AJ, Chaudhuri AA, Scherer F (2019). Determining cell type abundance and expression from bulk tissues with digital cytometry. Nat Biotechnol.

[31] Huang C, Zheng Y, Bai J, Shi C, Shi X, Shan H (2021). Hepatocyte growth factor overexpression promotes osteoclastogenesis and exacerbates bone loss in CIA mice. J Orthop Translat.

[32] Grandaunet B, Syversen SW, Hoff M, Sundan A, Haugeberg G, Van Der Heijde D (2011). Association between high plasma levels of hepatocyte growth factor and progression of radiographic damage in the joints of patients with rheumatoid arthritis. Arthritis Rheum.

[33] Gaffen SL (2009). Structure and signalling in the IL-17 receptor superfamily. Nat Rev Immunol.

[34] Chamberlain ND, Vila OM, Volin MV, Volkov S, Pope RM, Swedler W (2012). TLR5; a novel and unidentified inflammatory mediator in Rheumatoid Arthritis that correlates with disease activity score and joint TNF-α levels. J Immunol.

[35] Iwasaki Y, Takeshima Y, Fujio K (2020). Basic mechanism of immune system activation by mitochondria. Immunol Med.

[36] Chávez MD, Tse HM (2021). Targeting mitochondrial-derived reactive oxygen species in T Cell-Mediated autoimmune diseases. Front Immunol.

[37] McGarry T, Hanlon MM, Marzaioli V, Cunningham CC, Krishna V, Murray K (2021). Rheumatoid arthritis CD14+ monocytes display metabolic and inflammatory dysfunction, a phenotype that precedes clinical manifestation of disease. Clin Transl Immunology.

[38] Roszkowski L, Ciechomska M (2021). Tuning monocytes and macrophages for personalized therapy and diagnostic challenge in rheumatoid arthritis. Cells.

[39] Buch MH, Eyre S, McGonagle D (2021). Persistent inflammatory and non-inflammatory mechanisms in refractory rheumatoid arthritis. Nat Rev Rheumatol.

