# Supplementary material for: Monocyte-derived transcriptomes explain the ineffectiveness of abatacept in rheumatoid arthritis
Source: Arthritis Res Ther. 2024 Jan 2;26:1. doi: 10.1186/s13075-023-03236-y (PMC10759752; doi:10.1186/s13075-023-03236-y)
Supplement: Supplementary file 1 — Additional file 1: Supplementary Data S1. Interpretation of module 1 enrichment [file 13075_2023_3236_MOESM1_ESM.docx]

**Supplementary Data S1 Interpretation of module 1 enrichment**

We found the strongest enrichment in “module 1” (Figure 2C, Supplementary Table S5, original name: GOBP_CHEMOKINE_C_X_C_MOTIF_LIGAND_1_PRODUCTION). This module consisted of six genes (IL17A, IL17F, IL17RA, MYD88, TIRAP and TRPV4). Among these six genes, TIRAP, MYD88, and IL17RA were upregulated in non-responders (Figure 2A, Supplementary Table S3). TIRAP and MYD88 are reported to function as adapter proteins of IL-1 and all Toll-like receptors (TLRs) except for TLR3 [1–3]. Among IL-1 and such TLRs, only TLR5 was associated with response with nominal significance (*p* = 8.7×10^-3^, Supplementary Table S3). Then we calculated the inter-sample correlation between the four genes (TLR5, TIRAP, MYD88, IL17RA) and found all pairs except for the TLR5-TIRAP pair were significantly correlated (Supplementary Data Figure S1, Supplementary Table S6).


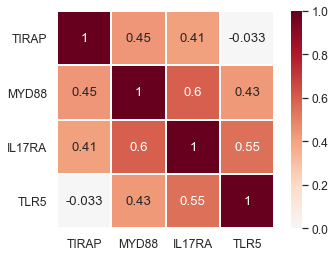


**Supplementary Data Figure S1.** Correlation between each gene’s expressions calculated from 44 specimens. The number in each box represents the Pearson's correlation coefficient. See also Supplementary Table S6.

Considering these results, TIRAP was not considered to be on the TLR5-MYD88 axis in the current specimens. Building upon these insights, we have concluded that a functional module encompassing TLR5, MYD88, and IL17RA is distinctly upregulated in non-responders to abatacept.

1. O’Neill LAJ, Bowie AG. The family of five: TIR-domain-containing adaptors in Toll-like receptor signalling. Nat Rev Immunol [Internet]. 2007 May [cited 2023 Aug 8];7(5):353–64. Available from: https://pubmed.ncbi.nlm.nih.gov/17457343/

2. Lannoy V, Côté-Biron A, Asselin C, Rivard N. TIRAP, TRAM, and Toll-Like Receptors: The Untold Story. Mediators Inflamm [Internet]. 2023 [cited 2023 Aug 8];2023. Available from: /pmc/articles/PMC10014160/

3. Guven-Maiorov E, Keskin O, Gursoy A, VanWaes C, Chen Z, Tsai CJ, et al. The Architecture of the TIR Domain Signalosome in the Toll-like Receptor-4 Signaling Pathway. Sci Rep [Internet]. 2015 Aug 21 [cited 2023 Aug 8];5. Available from: https://pubmed.ncbi.nlm.nih.gov/26293885/
