# Supplementary figures and images for: Monocyte-derived transcriptomes explain the ineffectiveness of abatacept in rheumatoid arthritis
Source: Arthritis Res Ther. 2024 Jan 2;26:1. doi: 10.1186/s13075-023-03236-y (PMC10759752; doi:10.1186/s13075-023-03236-y)

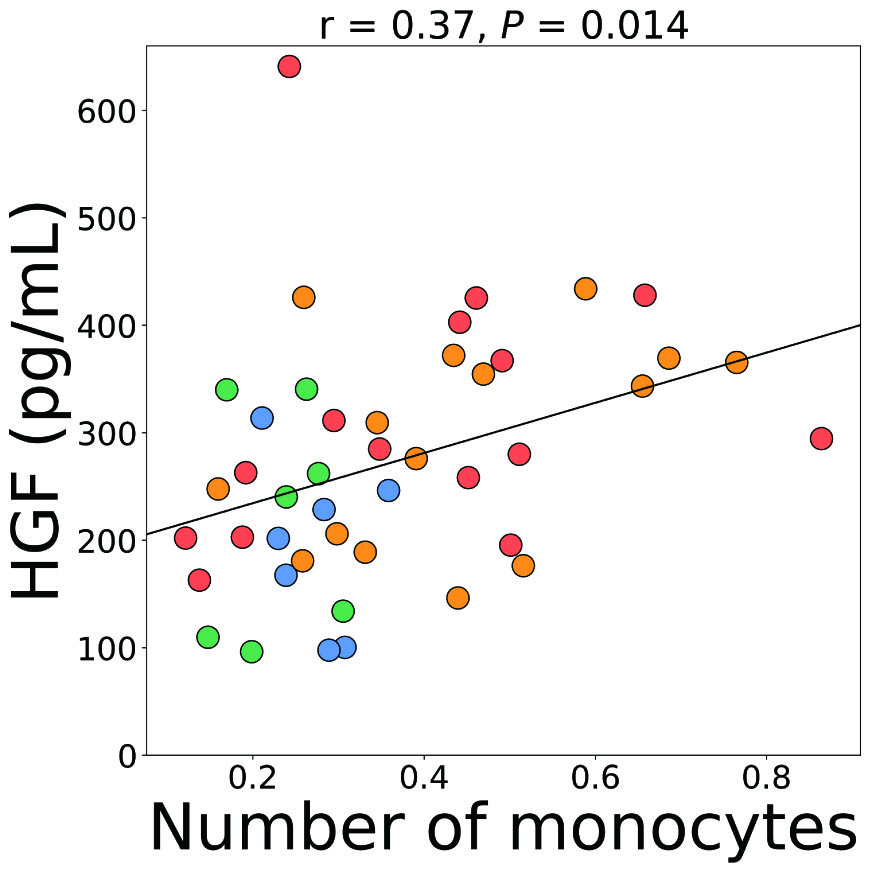

Supplement: Supplementary file 2 — Additional file 2: Fig. S1. Correlation between the number of monocytes and the level of HGF calculated in 44 specimens (22 individuals × 2 time points). The vertical line represents the number of monocytes in the peripheral blood (×103/μL). The dots indicate the specimens before treatment of responders (blue) and non-responders (orange), 3 months after treatment of responders (green) and non-responders (red). The Pearson's correlation coefficient (r), and the P-value calculated from linear regression are shown. [file 13075_2023_3236_MOESM2_ESM.jpg]

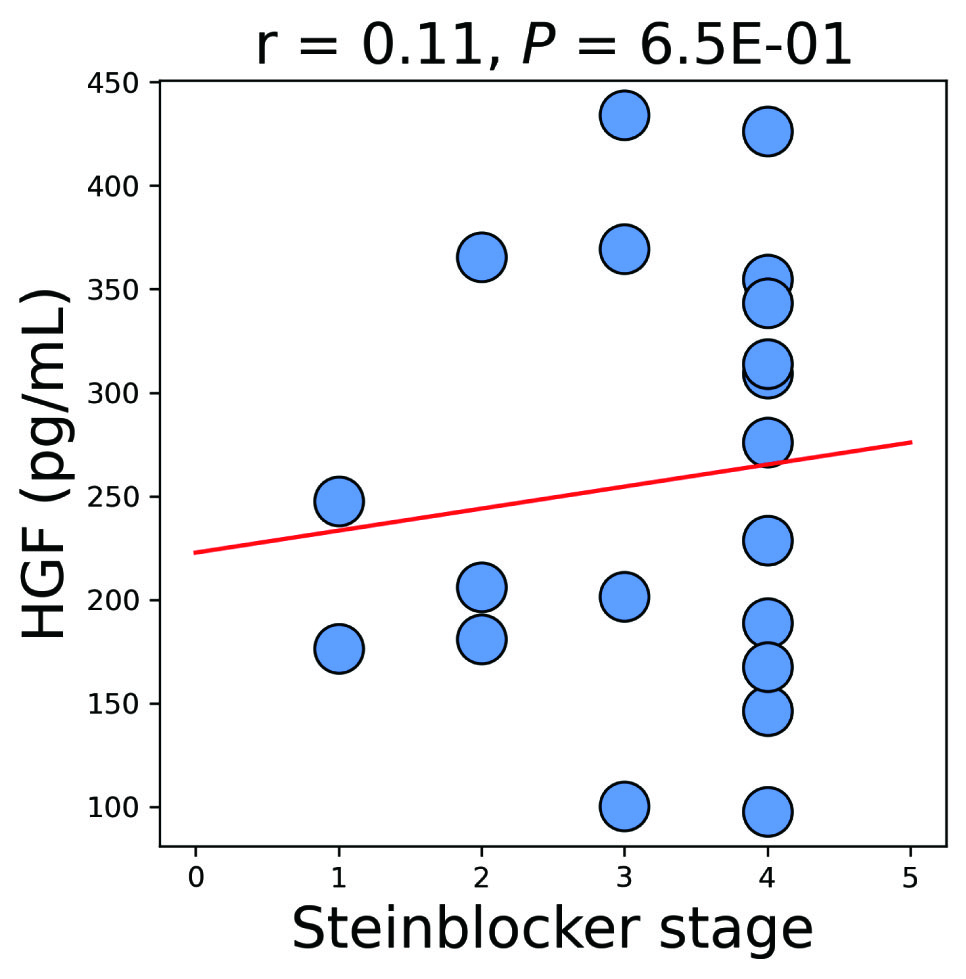

Supplement: Supplementary file 3 — Additional file 3: Fig. S2. Correlation between the Steinblocker stage and the levels of HGF before treatment calculated in 22 individuals. The Pearson's correlation coefficient (r), and the P-value calculated from linear regression are shown. [file 13075_2023_3236_MOESM3_ESM.jpg]

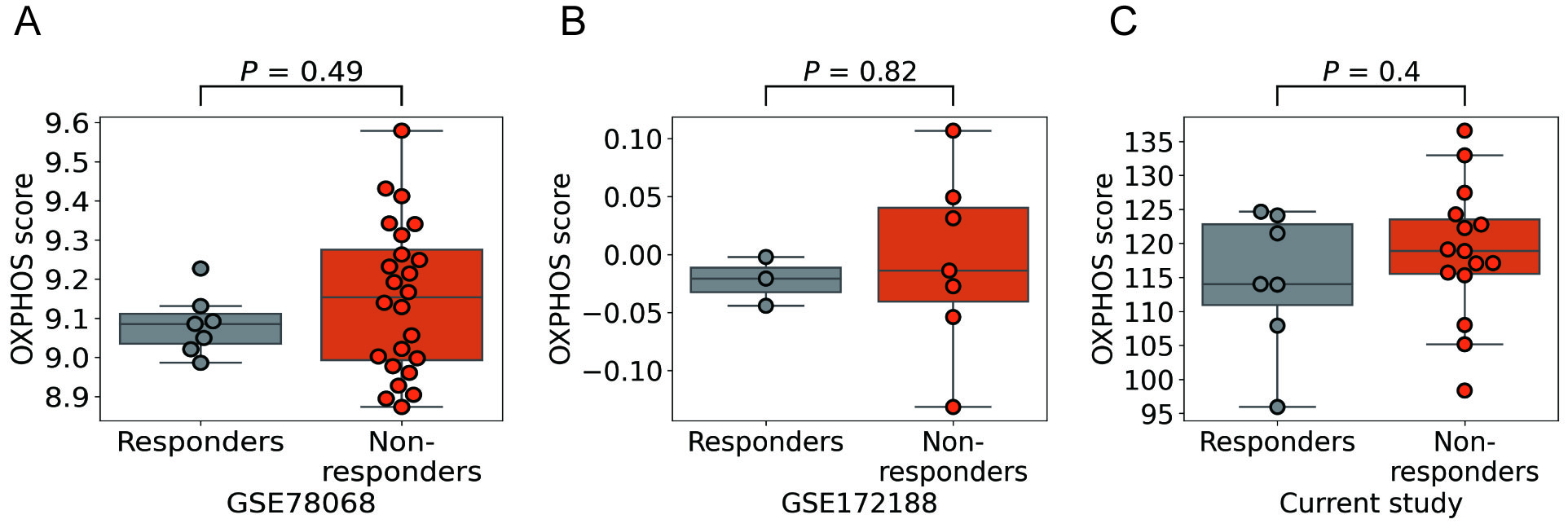

Supplement: Supplementary file 4 — Additional file 4: Fig. S3. Box plots comparing the OXPHOS scores between responders and non-responders in GSE78068 (A), GSE172188 (B), and the current study (C). The P-values calculated by the Mann-Whitney U test are shown. [file 13075_2023_3236_MOESM4_ESM.jpg]
